# Analysis of GNSS-RTK Monitoring Background Noise Characteristics Based on Stability Tests

**DOI:** 10.3390/s25020379

**Published:** 2025-01-10

**Authors:** Wencong Qi, Feilong Li, Lina Yu, Lilong Fan, Kai Zhang

**Affiliations:** 1 School of Mechanical Engineering, Tianjin University, Tianjin 300072, China; 2015201078@tju.edu.cn; 2 Centre for Artificial Intelligence and Robotics (CAIR), Hong Kong Institute of Science and Innovation, Chinese Academy of Sciences, Hong Kong, China; feilong.li@connect.polyu.hk; 3 Institute of Ocean Energy and Intelligent Construction, Tianjin University of Technology, Tianjin 300384, China; 4 China Railway Construction Bridge Engineering Bureau Group Co., Ltd., Tianjin 300300, China; 15822600521@163.com (L.F.); zkqd@163.com (K.Z.)

**Keywords:** GNSS-RTK, background noise, environmental effects, stability test

## Abstract

GNSS-RTK offers numerous advantages and broad prospects in structural dynamic monitoring in civil engineering. However, in practical applications, GNSS-RTK accuracy is susceptible to the monitoring environments, causing actual monitoring accuracy to fall below its calibrated accuracy. This study investigates the monitoring accuracy and spectral characteristics of GNSS-RTK based on stability tests under different environments related to reflection and obstruction conditions (i.e., concrete, grass, an obstructed balcony, and a water area). The findings indicate that in open environments of grass, concrete, and water, the standard deviation (STD) of GNSS-RTK monitored displacement is below 8 mm, its accuracy meeting the specifications of structural health monitoring. In the obstructed balcony environments, GNSS-RTK signals exhibit amplitude jumps, resulting in lower accuracy; however, during non-jump intervals, the STD of monitored displacement is below 10 mm, satisfying the structural health monitoring accuracy requirements. Moreover, the amplitudes of GNSS-RTK displacements in the concrete, grass, and water areas are basically consistent with the calibration accuracy of ±10 mm in the horizontal direction and ±20 mm in the elevation direction, while the amplitudes of GNSS-RTK displacements in the obstructed balcony condition are far greater than the calibration accuracy. The spectral analysis of GNSS-RTK signals reveals that multipath errors in concrete, grass, and obstructed balcony environments are primarily concentrated in the low-frequency range within 0.04 Hz, while the internal white noise of the instrument is widely and evenly distributed across the whole frequency domain. Based on these findings, adaptive methods, such as filter methods and multipath error correction techniques, are proposed for the de-noising of GNSS-RTK background noise.

## 1. Introduction

The GNSS-RTK technology, with its long measurement range, supports differential positioning within a 10 km signal range, and is virtually unrestricted by distance. It offers high automation and robust environmental adaptability, and enables real-time monitoring under all weather conditions. Consequently, the GNSS-based monitoring of large-scale civil engineering structures, such as long-span bridges, super high-rise buildings, and dams, holds numerous advantages and broad prospects for application [1,2,3]. In particular, the utilization of GPS, BDS, and other multi-systems enables an augmented quantity of satellites and geometric structures in comparison to singular GPS monitoring. This enhancement contributes to the refinement of the availability, reliability, and monitoring accuracy of GNSS-RTK. Such advancements prompt a wide application in structural dynamic deformation monitoring [4,5].

GNSS monitoring is carried out under the influence of multiple errors, including satellite error, receiver and station error, signal propagation error, etc. The difference calculation process has the capability to eliminate satellite correlation error, receiver clock discrepancy, ionospheric and tropospheric delays, and other related factors. However, the multipath error cannot be eliminated by the difference method due to a lack of correlation between the monitoring station and the base station [6,7,8,9,10]. As a result, the influence of the multipath effect on the observed carrier phase can reach several centimeters at most, which has become the main error source restricting the improvement of GNSS real-time deformation monitoring accuracy [11]. Research indicates that multipath error is dependent on the phase delay and reflection coefficient of the reflected signal. In addition, multipath error is closely related to the receiver’s surrounding environment, where obstructions intensify multipath effects [12,13,14,15,16]. Additionally, multipath errors correlate with satellite geometry, resulting in a periodic repetition [17,18,19]. Researchers have employed various methods to reduce multipath errors based on satellite- and signal-related characteristics, mainly including the following four methods. (1) From the perspective of satellites, the influence of multipath errors could be weakened by weighting observation values based on feature vectors such as carrier to noise ratio and elevation angle [20,21,22,23,24]. (2) The post processing of coordinate domain time series using signal filtering and denoising algorithms can also eliminate the multipath effects [25,26,27]. (3) The sidereal filtering method, based on the cyclic repetition characteristic of multipath signals, can extract multipath error models and correct them by calculating the orbital repetition period of each satellite [28,29,30,31,32]. (4) Based on the repetitive characteristics of multipath spatial domains, the sky is divided into multipath semi spherical maps at equal angular intervals to identify and reduce multipath errors [33].

**Figure 1:**
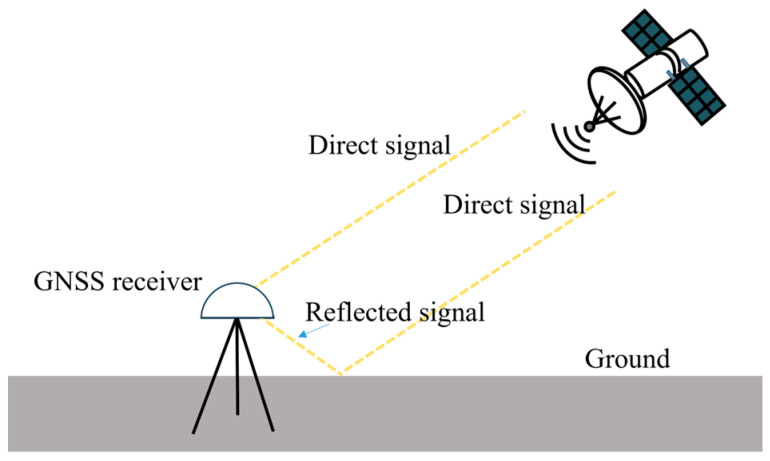
Principle of multipath error.

The principle of multipath error is given in Figure 1. The simultaneous reception of both direct and reflected signals by the GNSS receiver leads to the multipath error. In practical structural monitoring, the multipath error varies in different environments, and the curtain walls, water surfaces, and other strong reflectors would aggravate the multipath effect. Given the diversity of monitoring structures and the complexity of their surrounding environments, it is essential to explore GNSS monitoring accuracy across various environments, while concurrently analyzing the spectral characteristics of GNSS-RTK, thereby achieve the effective separation of multipath errors from structural dynamic deformation signals.

To assess the dynamic monitoring accuracy and background noise characteristics of GNSS-RTK in different monitoring environments, four typical monitoring conditions, open concrete, grass, an obstructed balcony, and a water area, were selected for stability tests. In theory, the three-dimensional coordinate value of the monitoring point should be fixed, but the absolute position coordinates of the monitoring point change with time due to the influence of multipath error and internal observation noise. Therefore, the data from stability tests can be employed for background noise characteristics analysis. This paper is structed as follows: Section 2 covers experimental design; Section 3 addresses data processing methods; Section 4 conducts data analysis; and Section 5 summarizes the main conclusions.

## 2. Experimental Design

Based on GNSS-RTK real-time dynamic difference technology, the three-dimensional geographical coordinate value of the point can be obtained directly. The relative displacement of GNSS-RTK is obtained by coordinate transformation and the removal of the mean value. Under the premise of uniform equipment modulation and unified difference mode, the variation difference of the displacement time-history curve obtained in different environments is mainly due to the multipath effect. This experiment aims to verify the stability of the GNSS receiver in different environments, comparatively analyze the impact of environment-induced multipath effects on monitoring accuracy, and further investigate the time-frequency characteristics of GNSS-RTK background noise in different environments.

**Figure 2:**
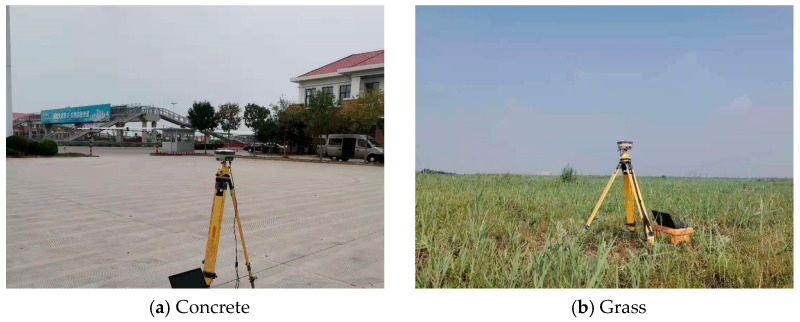

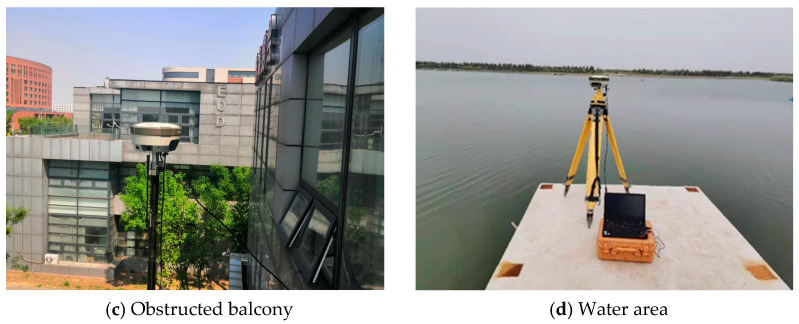
GNSS-RTK stability test environments.

The experimental site is located in the Binhai New Area of Tianjin, where monitoring stations were set up in three environments: concrete, grass, and an obstructed balcony, as shown in Figure 2. During the experimental procedures, all GNSS receivers were uniformly set up to function in a tri-constellation mode encompassing GPS, BDS, and GLONASS. The configuration included a cut-off angle of 15° and a sampling rate of 10 Hz. The calibration precision of the GNSS real-time kinematic (RTK) system is ±10 mm horizontally and ±20 mm vertically. Given that internal observational noise is solely related to the monitoring equipment itself, under consistent device configuration and differential calculation, any variations in displacement obtained from different environments can be primarily attributed to multipath effects. To avoid the influence of accidental factors and ensure result reliability, five sets of measured data, each with a duration of 1200 s, were collected and analyzed in each environment.

## 3. Data Processing Methods

### 3.1. Signal Evaluation Metrics

The standard deviation (STD) reflects the dispersion of the signal; the smaller the standard deviation, the better the signal stability. The STD value is expressed as follows:

(1) $$S=\sqrt{\frac{\sum_{t=1}^{N}{\left(y(t)-\bar{y}\left(t\right)\right)}^{2}}{N}}$$

where *t* represents the time variable; y(t) represents the original signal; *N* indicates the signal length; and $\bar{y}\left(t\right)$ represents the mean value of the original signal.

### 3.2. Normalized Power Spectral Density Calculation

The power spectral density (PSD) can reveal the distribution of various frequency components in a signal, which is an important parameter for describing the random vibration characteristics of structures. By observing the peak values in the amplitude-frequency curve of the PSD, one can intuitively assess the purity of the signal and approximate the structure’s natural frequency. The normalized power spectral density (NPSD) [22] could reflect the energy distribution of the signal in the frequency domain, through which the natural frequency of the vibration can be identified. The NPSD can be expressed as follows:

(2) $$P(f)=\frac{[a^{2}(f)+b^{2}(f)-{\sigma^{2}}_{stat}/n]T}{{F^{2}}_{av}}$$

(3) $$a(f)=\frac{1}{n}\sum_{j=0}^{n-1}F_{j}\cos(2\pi ft_{j})$$

(4) $$b(f)=\frac{1}{n}\sum_{j=0}^{n-1}F_{j}\sin(2\pi ft_{j})$$

where $F_{j}$ is the source count rate at the time, $t_{j}(0<t_{j}<n-1)$ is the total time length, $F_{av}$ is the mean value of source count rates, and $\sigma_{stat}$ is the error due to counting statistics. The power P(f) for some discrete frequencies given by f=k/T ($\bar{k}$ is an integer and 1<k<n/2) can be calculated and averaged.

## 4. Data Analysis

### 4.1. Time Domain Analysis

**Figure 3:**
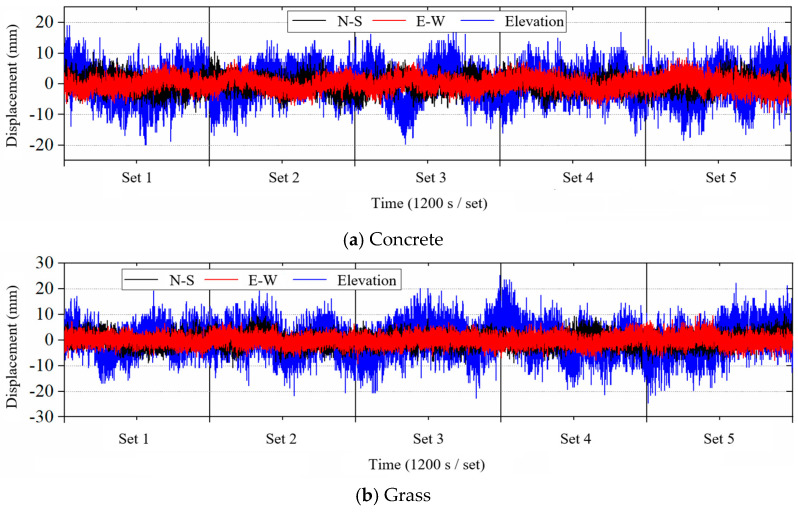

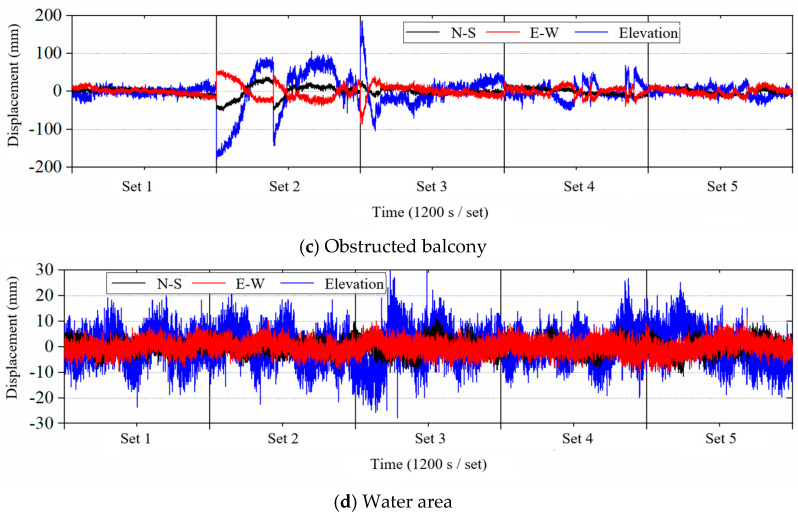
GNSS-RTK relative displacement time series.

The monitoring signal obtained by the GNSS-RTK difference calculation is the three-dimensional coordinate value in the World Geodetic System 1984 (WGS84) coordinate system. First, the monitoring signal needs to be processed by coordinate conversion. The absolute coordinate value in the WGS84 coordinate system is converted to the displacement value in the Cartesian coordinate system. Then, the displacement value is subtracted from the average to obtain the GNSS-RTK displacement time series. GNSS-RTK displacement time series in different environments are given in Figure 3. Overall, the GNSS-RTK series in the concrete, water, and grass environments remain relatively stable, while in the obstructed balcony environment, they exhibit multiple instances of amplitude jumps and drifts. Additionally, the amplitude of GNSS-RTK series in the north–south and east–west directions is smaller than in the elevation direction, indicating that monitoring accuracy in the horizontal direction is higher than in the vertical direction.

Specifically, the displacement amplitude of the monitoring signal primarily ranges within ±10 mm horizontally and ±20 mm in the elevation direction in the concrete, water, and grass environments, aligning with the instrument’s calibrated accuracy and demonstrating the reliability of GNSS-RTK monitoring. In the obstructed balcony environment, the GNSS-RTK monitoring signals for the 2~4 sets show significant amplitude jumps, particularly severe in the elevation direction, with values predominantly distributed within ±200 mm, which does not meet the specifications of structural monitoring requirements. In contrast, the first and fifth sets of monitoring signals remain relatively stable, with horizontal displacement amplitudes mostly within ±20 mm and elevation amplitudes around ±30 mm. The instability of the monitoring signal in the obstructed balcony environment results from the building obstruction, which significantly reduces the number of visible satellites and adversely affects differential positioning.

The STD value of GNSS-RTK displacement series in N–S, E–W, and elevation directions are summarized in Table 1, Table 2 and Table 3. Overall, the STD values of the displacement series in the N–S and E–W directions are consistently lower than in the elevation direction, which aligns with the amplitude analysis results shown in Figure 3. According to China’s “Code for Deformation Measurement of Buildings” (JGJ8-2016) [34], the standard deviation for wind-induced vibration deformation measurements should not exceed 10 mm. In concrete, water, and grass environments, the STD values of the displacement time series are generally below 3 mm in the horizontal directions and 8 mm in the elevation direction. In the obstructed balcony environment, the STD values of the first dataset are all below 10 mm, while in the fifth dataset, the STD is below 10 mm in the horizontal direction and 11.26 mm in the elevation direction. The GNSS-RTK monitored signal in the obstructed balcony environment is more unstable, though the horizontal direction performs better than the elevation direction.

The amplitude values of GNSS-RTK displacement series in N–S, E–W, and elevation directions are summarized in Table 4, Table 5 and Table 6. The amplitude values of the displacement series in N–S and E–W directions are consistently lower than in the elevation direction, which aligns with the analysis results shown in Figure 3 and Table 1, Table 2 and Table 3. Moreover, the amplitude values of the displacement in the horizontal direction are basically the same as the calibration accuracy of the GNSS equipment. Although the amplitude values of GNSS-RTK in the obstructed balcony condition in the horizontal direction perform better than those of the elevation direction, all of its results are far greater than the calibration accuracy. The number of visible satellites and PDOP of GNSS-RTK in different monitoring scenarios are shown in Table 7 and Table 8, respectively. In general, the mean values of visible satellites of GNSS-RTK range from 11 to 13, which can meet the needs of dynamic deformation monitoring. However, it should be noted that the geometric accuracy of the satellites is slightly large in the obstructed balcony condition. Especially in the second and third periods, there are frequent jumps for both displacement and PDOP in the time domain. Therefore, it is essential to reduce environmental interference during the implementation of GNSS-RTK-based structural dynamic monitoring for optimal outcomes. Moreover, since the monitoring location is fixed and its surrounding environment is usually difficult to alter, the filtering and de-noising process should account for these adverse effects.

### 4.2. Frequency Domain Analysis

**Figure 4:**
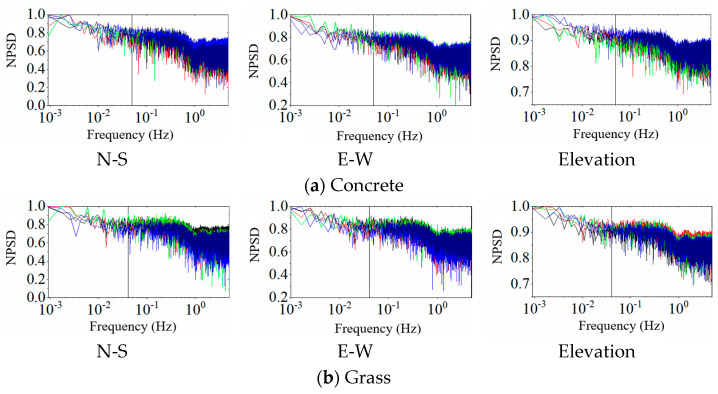

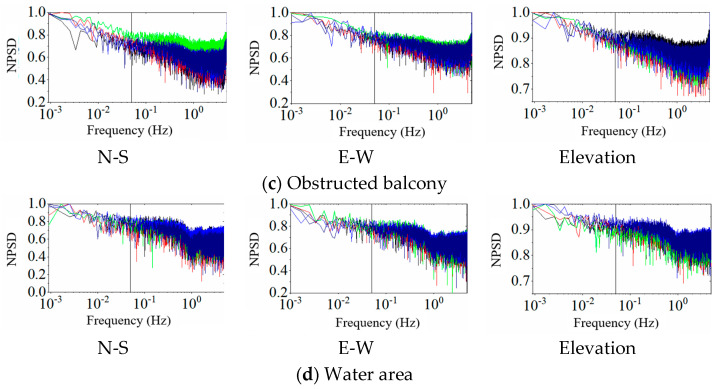
NPSD Functions.

To investigate the spectral characteristics of GNSS-RTK background noise in different environments, the NPSD function of GNSS-RTK is calculated, as shown in Figure 4. For ease of comparative analysis, the frequency values on the X-axis were logarithmically transformed, and the five datasets for each environment and direction were superimposed. In the low-frequency region, the multipath error is dominant and the noise energy is relatively high, indicating that the multipath error has a significant impact on GNSS-RTK monitoring results. In contrast, internal white noise from the instrument is widely distributed across the whole frequency domain, with relatively uniform noise energy. Specifically, in the concrete, grass, and obstructed balcony environments, the low frequency and high energy multipath error is primarily concentrated within 0.04 Hz. Therefore, when the energy of low-frequency noise is high and the fundamental vibration frequency of the monitored structure is much higher than 0.04 Hz, the filter method can be used to de-noise low-frequency noise components, such as Chebyshev and Butterworth methods. Additionally, model correction methods can be used to mitigate multipath error based on its periodic repetition, such as independent component analysis (ICA) and principal component analysis (PCA) methods. For the interference of internal white noise, separation methods based on white noise properties can be considered for de-noising, such as the empirical mode decomposition (EMD) and wavelet transform methods.

## 5. Conclusions

GNSS-RTK is widely used in the dynamic monitoring of large civil engineering structures. However, its stability and accuracy are susceptible to environmental influences. To investigate GNSS-RTK monitoring accuracy and the spectral characteristics of back-ground noise in various environments, stability tests were conducted in four settings, concrete, grass, a water area and an obstructed balcony, leading to the following conclusions:

(1) In open environments of grass, concrete, and water, the displacement amplitudes of GNSS-RTK primarily fall within ±10 mm in the horizontal direction and ±20 mm in the elevation direction, with standard deviation (STD) values of no more than 8 mm. It is confirmed that GNSS-RTK is suitable for monitoring the dynamic structural deformation under environmental excitation.

(2) In obstructed environments, the signal exhibits multiple amplitude jumps, indicating lower monitoring stability; however, during non-jump intervals, it marginally meets dynamic monitoring specifications. Therefore, when using GNSS-RTK for monitoring, obstructed environments should be avoided whenever possible. When obstruction is unavoidable, the methods for filtering and de-noising should account for these adverse effects.

(3) In both open and obstructed environments, the multipath error of GNSS-RTK is mainly concentrated around the low-frequency range of 0.04 Hz with high energy amplitudes, while the internal white noise of the instrument is widely and uniformly distributed across the frequency domain. Accordingly, the multipath error can be filtered out using the filter method when the frequency domain of multipath error does not overlap with the natural frequency of the structure. Additionally, model correction methods can be used to extract the multipath error based on its periodic repetition characteristics, while internal white noise can be eliminated based on its inherent characteristics.

## Figures and Tables

**Table 1:** STD values of GNSS-RTK displacement series in the N–S direction (mm).

Environmental Characteristics	Test Grouping					Mean Value
	1	2	3	4	5	
Concrete	2.54	2.57	2.51	2.32	2.34	2.46
Grass	2.39	2.61	2.21	2.50	2.95	2.53
Obstructed balcony	6.94	21.39	6.27	8.26	6.42	9.86
Water area	2.22	2.24	3.27	2.50	2.82	2.61

**Table 2:** STD values of GNSS-RTK displacement series in the E–W direction (mm).

Environmental Characteristics	Test Grouping					Mean Value
	1	2	3	4	5	
Concrete	1.99	2.15	1.90	2.08	2.46	2.12
Grass	1.80	1.91	1.89	2.09	2.30	2.00
Obstructed balcony	7.97	23.54	16.12	11.58	9.33	13.71
Water area	2.44	2.54	2.60	2.60	2.83	2.60

**Table 3:** STD values of GNSS-RTK displacement series in the elevation direction (mm).

Environmental Characteristics	Test Grouping					Mean Value
	1	2	3	4	5	
Concrete	5.35	4.63	5.58	4.39	5.25	5.04
Grass	5.02	5.39	6.23	6.20	6.20	5.81
Obstructed balcony	9.01	75.01	36.92	20.63	11.26	30.56
Water area	5.80	6.09	7.34	5.79	6.56	6.32

**Table 4:** Amplitude values of GNSS-RTK displacement series in the N–S direction (mm).

Environmental Characteristics	Test Grouping					Mean Value
	1	2	3	4	5	
Concrete	15.50	17.88	23.15	17.35	21.01	23.15
Grass	17.07	20.44	17.87	20.44	19.60	19.09
Obstructed balcony	38.88	90.91	45.48	42.70	34.18	50.43
Water area	15.50	17.88	23.15	17.35	21.01	18.98

**Table 5:** Amplitude values of GNSS-RTK displacement series in the E–W direction (mm).

Environmental Characteristics	Test Grouping					Mean Value
	1	2	3	4	5	
Concrete	18.07	19.14	19.50	19.72	21.04	21.25
Grass	14.56	13.58	16.33	14.93	18.45	15.57
Obstructed balcony	49.29	96.54	126.39	68.66	53.82	78.94
Water area	18.07	19.14	19.50	19.72	21.04	19.49

**Table 6:** Amplitude values of GNSS-RTK displacement series in the elevation direction (mm).

Environmental Characteristics	Test Grouping					Mean Value
	1	2	3	4	5	
Concrete	44.00	47.00	61.00	47.00	45.00	61.00
Grass	36.00	41.00	48.00	45.00	47.00	43.40
Obstructed balcony	67.00	286.00	291.00	120.00	78.00	168.40
Water area	44.00	47.00	61.00	47.00	45.00	48.80

**Table 7:** The number of visible satellites (mean value) of GNSS-RTK.

Environmental Characteristics	Test Grouping					Mean Value
	1	2	3	4	5	
Concrete	13	13	14	13	12	13
Grass	13	14	12	13	13	13
Obstructed balcony	12	11	10	12	13	12
Water area	12	11	10	11	11	11

**Table 8:** The PDOP value of GNSS-RTK.

Environmental Characteristics	Test Grouping					Mean Value
	1	2	3	4	5	
Concrete	0.9361	0.8561	0.7836	0.7508	0.8146	0.8282
Grass	0.8006	0.7665	0.9036	0.8323	0.8073	0.8221
Obstructed balcony	1.0245	1.1229	1.1637	1.0030	0.9649	1.0358
Water area	0.9354	1.0303	1.0810	0.9545	0.9329	1.0068

## Data Availability

Data is availability on request.

## References

[1] Xi R., Zhou X., Jiang W., Chen Q. (2018). Simultaneous estimation of dam displacements and reservoir level variation from GPS measurements. Measurement.

[2] Yu J., Meng X., Yan B., Xu B., Fan Q., Xie Y. (2020). Global Navigation Satellite System-based positioning technology for structural health monitoring: A review. Struct. Control. Health Monit..

[3] Yu L., Xiong C., Chen W., Gao Y., Ye Z., Shi Q. (2021). A combined algorithm for denoising GNSS-RTK positioning solutions with application to displacement monitoring of a super-high-rise building. Meas. Sci. Technol..

[4] Kim J., Kim K., Sohn H. (2014). Autonomous dynamic displacement estimation from data fusion of acceleration and intermittent displacement measurements. Mech. Syst. Signal Process..

[5] Xu Y., Brownjohn J.M.W., Hester D., Koo K.Y. (2017). Long-span bridges: Enhanced data fusion of GPS displacement and deck accelerations. Eng. Struct..

[6] Zheng F., Li Q., Wang J., Gong X., Jia H., Zhang C., Shi C. (2024). GNSS NLOS detection method based on stacking ensemble learning and applications in smartphones. GPS Solut..

[7] Xu B., Jia Q., Luo Y., Hsu L.-T. (2019). Intelligent GPS L1 LOS/multipath/NLOS classifiers based on correlator-, RINEX-and NMEA-level measurements. Remote Sens..

[8] Xia Y., Pan S., Meng X., Gao W., Wen H. (2020). Robust Statistical Detection of GNSS Multipath Using Inter-Frequency C/N0 Differences. Remote Sens..

[9] Li L., Xu Z., Jia Z., Lai L., Shen Y. (2024). An efficient GNSS NLOS signal identification and processing method using random forest and factor analysis with visual labels. GPS Solut..

[10] Li X., Wang B., Li X., Huang J., Lyu H., Han X. (2022). Principle and performance of multi-frequency and multi-GNSS PPP-RTK. Satell. Navig..

[11] Yu J., Xie Y., Fang Z., Peng Z., Yang R., Wang Y. (2024). Identification of bridge modal parameters from GNSS data by integrating IEWT and robust ICA algorithm. Meas. Sci. Technol..

[12] Wang J., Wang J., Roberts C. (2009). Reducing GPS carrier phase errors with EMD-wavelet for precise static positioning. Surv. Rev. Dir. Overseas Surv..

[13] Xiong C., Wang M., Shang Z., Liu T., Shi Q. (2023). Modal frequencies evaluation of a damaged bridge using RCVMD algorithm based on sensor dynamic responses. Meas. Sci. Technol..

[14] Tao Y., Liu C., Liu C., Zhao X., Hu H. (2021). Empirical wavelet transform method for GNSS coordinate series denoising. J. Geovisualization Spat. Anal..

[15] Li W., Guo J. (2024). Extraction of periodic signals in Global Navigation Satellite System (GNSS) vertical coordinate time series using the adaptive ensemble empirical modal decomposition method. Nonlinear Process. Geophys..

[16] Park J.C., Gil H.B., Kang S.G., Lim C.W. (2010). Dynamic characteristics of a cable-stayed bridge using global navigation satellite system. KSCE J. Civ. Environ. Eng. Res..

[17] Nath S., Chetia B., Kalita S. (2023). Ionospheric TEC prediction using hybrid method based on ensemble empirical mode decomposition (EEMD) and long short-term memory (LSTM) deep learning model over India. Adv. Space Res..

[18] Hoar G., Inglis D., MacInnis M., Tobin S. An Autonomous GNSS Wave Sensor Module for Deployment on Existing Buoy Infrastructure: Comparison and Validation of Co-Located GNSS and Accelerometer Directional Wave Sensors. Proceedings of the 2019 IEEE/OES Twelfth Current, Waves and Turbulence Measurement (CWTM).

[19] Xing L., Wen Y., Thomas DW P., Zhang J., Zhang D., Xiao J. A Joint time-frequency analytical method for electromagnetic interference in railway GNSS system. Proceedings of the 2020 International Symposium on Electromagnetic Compatibility-EMC EUROPE.

[20] Zhong P., Ding X., Yuan L., Xu Y., Kwok K., Chen Y. (2010). Sidereal filtering based on single differences for mitigating GPS multipath effects on short baselines. J. Geod..

[21] Dong D., Wang M., Chen W., Zeng Z., Song L., Zhang Q., Cai M., Cheng Y., Lv J. (2016). Mitigation of multipath effect in GNSS short baseline positioning by the multipath hemispherical map. J. Geod..

[22] Ye S., Chen D., Liu Y., Jiang P., Tang W., Xia P. (2015). Carrier phase multipath mitigation for BeiDou navigation satellite system. GPS Solut..

[23] Špánik P., Hefty J. (2017). Multipath detection with the combination of SNR measurements—Example from urban environment. Geod. Cartogr..

[24] Ng H.F., Zhang G., Yang K.Y., Yang S.X., Hsu L.T. (2020). Improved weighting scheme using consumer-level GNSS L5/E5a/B2a pseudorange measurements in the urban area. Adv. Space Res..

[25] Chen W., Xiong C., Yu L., Lian S., Ye Z. (2021). Dynamic monitoring of an offshore jacket platform based on RTK-GNSS measurement by CF-CEEMDAN method. Appl. Ocean. Res..

[26] Xiong C., Wang M., Chen W. (2022). Data analysis and dynamic characteristic investigation of large-scale civil structures monitored by RTK-GNSS based on a hybrid filtering algorithm. J. Civ. Struct. Health Monit..

[27] Azarbad R.M., Mosavi R.M. (2014). A new method to mitigate multipath error in single-frequency GPS receiver with wavelet transform. GPS Solut..

[28] Agnew C.D., Larson M.K. (2007). Finding the repeat times of the GPS constellation. GPS Solut..

[29] Choi K., Bilich A., Larson K.M., Axelrad P. (2004). Modified sidereal filtering: Implications for high-rate GPS positioning. Geophys. Res. Lett..

[30] Atkins C., Ziebart M. (2016). Effectiveness of observation-domain sidereal filtering for GPS precise point positioning. GPS Solut..

[31] Wang M., Wang J., Dong D., Li H., Han L., Chen W. (2018). Comparison of Three Methods for Estimating GPS Multipath Repeat Time. Remote Sens..

[32] Wang M., Wang J., Dong D., Chen W., Li H., Wang Z. (2018). Advanced Sidereal Filtering for Mitigating Multipath Effects in GNSS Short Baseline Positioning. ISPRS Int. J. Geo-Inf..

[33] Wang Z., Chen W., Dong D., Wang M., Cai M., Yu C., Zheng Z., Liu M. (2019). Multipath mitigation based on trend surface analysis applied to dual-antenna receiver with common clock. GPS Solut..

[34] (2016). Code for Deformation Measurement of Buildings.

